# Identification of Recent Tuberculosis Exposure Using QuantiFERON-TB Gold Plus, a Multicenter Study

**DOI:** 10.1128/Spectrum.00972-21

**Published:** 2021-11-10

**Authors:** Sandra Pérez-Recio, Natàlia Pallarès, Maria D. Grijota-Camino, Adrián Sánchez-Montalvá, Laura Barcia, Silvia Campos-Gutiérrez, Virginia Pomar, Ramón Rabuñal-Rey, María Elvira Balcells, Deniz Gazel, Natalia Montiel, Diego Vicente, Ivana Goić-Barišić, Thomas Schön, Jakob Paues, Ivana Mareković, Juana Cacho-Calvo, Aleksandra Barac, Delia Goletti, Mercedes García-Gasalla, José María Barcala, María Teresa Tórtola, Luis Anibarro, Isabel Suárez-Toste, Esther Moga, María J. Gude-Gonzalez, Rodrigo Naves, Tekin Karslıgil, Tania Martin-Peñaranda, Goran Stevanovic, Matilde Trigo, Verónica Rubio, İlkay Karaoğlan, Nazan Bayram, Fernando Alcaide, Cristian Tebé, Miguel Santin

**Affiliations:** a Tuberculosis Unit, Department of Infectious Diseases, Bellvitge University Hospital -Bellvitge Biomedical Research Institute (IDIBELL), L’Hospitalet de Llobregat, Barcelona, Spain; b Biostatistics Unit (UBiDi), Bellvitge Biomedical Research Institute (IDIBELL), L’Hospitalet de Llobregat, Barcelona, Spain; c Department of Infectious Diseases, PROSICS, Vall d'Hebrón University Hospital, Barcelona, Spain; d Tuberculosis Unit, Department of Internal Medicine, Complexo Hospitalario Universitario de Pontevedra, Pontevedra, Spain; e Microbiology and Infection Control Service, University Hospital of Canary Islands, Tenerife, Canary Islands, Spain; f Infectious Diseases Unit, Department of Internal Medicine, Hospital de la Santa Creu i Sant Pau, Barcelona, Spain; g Infectious Diseases Unit, Department of Internal Medicine, Hospital Lucus Augusti, Lugo, Spain; h Department of Infectious Diseases, School of Medicine, Pontificia Universidad Católica de Chile, Santiago, Chile; i Department of Medical Microbiology, Faculty of Medicine, Gaziantep University, Gaziantep, Turkey; j Department of Microbiology, Hospital Costa del Sol, Marbella, Málaga, Spain; k Department of Microbiology, Hospital Universitario Donostia, San Sebastian, Spain; l Department of Clinical Microbiology, University Hospital of Split, Split, Croatia; m School of Medicine, University of Split, Split, Croatia; n Department of Infectious Diseases, Kalmar County Hospital, Kalmar, Linköping University, Sweden; o Division of Infectious Diseases, Department of Biomedical and Clinical Sciences, Linköping University, Linköping, Sweden; p Department for Clinical and Molecular Microbiology, University Hospital Centre Zagreb, Zagreb, Croatia; q Department of Microbiology, Getafe University Hospital, Getafe, Madrid, Spain; r Clinic for Infectious and Tropical Diseases, Clinical Center of Serbia, Belgrade, Serbia; s Faculty of Medicine, University of Belgrade, Belgrade, Serbia; t Istituto Nazionale per le Malattie Infettive Lazzaro Spallanzani-IRCCS, Rome, Italy; u Infectious Diseases Unit, Department of Internal Medicine, Hospital Universitari Son Llàtzer-IdISBa, Palma de Mallorca, Illes Balears, Spain; v Infectious Diseases Unit, Hospital Universitario de Jerez, Jerez de la Frontera, Cádiz, Spain; w Department of Microbiology, Vall d’Hebrón University Hospital, Barcelona, Spain; x Department of Genetics and Microbiology, Universitat Autònoma de Barcelona, Barcelona, Spain; y Pneumology Service, University Hospital of Canary Islands, Tenerife, Canary Islands, Spain; z Department of Immunology, Hospital de la Santa Creu i Sant Pau, Biomedical Research Institute Sant Pau (IIB Sant Pau), Barcelona, Spain; aa Department of Microbiology, Hospital Lucus Augusti, Lugo, Spain; bb Program of Immunology, Institute of Biomedical Sciences, Faculty of Medicine, Universidad de Chile, Santiago, Chile; cc Microbiology Department, Complexo Hospitalario Universitario de Pontevedra, Pontevedra, Spain; dd Department of Infectious Diseases and Clinical Microbiology, Faculty of Medicine, Gaziantep University, Gaziantep, Turkey; ee Department of Pulmonary Diseases, Faculty of Medicine, Gaziantep University, Gaziantep, Turkey; ff Department of Microbiology, Bellvitge University Hospital —Bellvitge Biomedical Research Institute (IDIBELL), L’Hospitalet de Llobregat, Barcelona, Spain; gg Department of Pathology and Experimental Therapy, University of Barcelona, L’Hospitalet de Llobregat, Barcelona, Spain; hh Department of Clinical Sciences, University of Barcelona, L’Hospitalet de Llobregat, Barcelona, Spain; Quest Diagnostics Nichols Institute

**Keywords:** QuantiFERON-TB gold plus, diagnosis, latent tuberculosis infection, tuberculosis-specific CD8 T cells

## Abstract

We investigated whether the difference of antigen tube 2 (TB2) minus antigen tube 1 (TB1) (TB2−TB1) of the QuantiFERON-TB gold plus test, which has been postulated as a surrogate for the CD8^+^ T-cell response, could be useful in identifying recent tuberculosis (TB) exposure. We looked at the interferon gamma (IFN-γ) responses and differences in TB2 and TB1 tubes for 686 adults with QFT-plus positive test results. These results were compared among groups with high (368 TB contacts), low (229 patients with immune-mediated inflammatory diseases [IMID]), and indeterminate (89 asylum seekers or people from abroad [ASPFA]) risks of recent TB exposure. A TB2−TB1 value >0.6 IU·ml^−1^ was deemed to indicate a true difference between tubes. In the whole cohort, 13.6%, 10.9%, and 11.2% of cases had a TB2>TB1 result in the contact, IMID, and ASPFA groups, respectively (*P* = 0.591). The adjusted odds ratios (aORs) for an association between a TB2−TB1 result of >0.6 IU·ml^−1^ and risk of recent exposure versus contacts were 0.71 (95% confidence interval [CI], 0.31 to 1.61) for the IMID group and 0.86 (95% CI, 0.49 to 1.52) for the ASPFA group. In TB contact subgroups, 11.4%, 15.4%, and 17.7% with close, frequent, and sporadic contact had a TB2>TB1 result (*P* = 0.362). The aORs versus the close subgroup were 1.29 (95% CI, 0.63 to 2.62) for the frequent subgroup and 1.55 (95% CI, 0.67 to 3.60) for the sporadic subgroup. A TB2−TB1 difference of >0.6 IU·ml^−1^ was not associated with increased risk of recent TB exposure, which puts into question the clinical potential as a proxy marker for recently acquired TB infection.

**IMPORTANCE** Contact tuberculosis tracing is essential to identify recently infected people, who therefore merit preventive treatment. However, there are no diagnostic tests that can determine whether the infection is a result of a recent exposure or not. It has been suggested that by using the QuantiFERON-TB gold plus, an interferon gamma (IFN-γ) release assay, a difference in IFN-γ production between the two antigen tubes (TB2 minus TB1) of >0.6 IU·ml^−1^ could serve as a proxy marker for recent infection. In this large multinational study, infected individuals could not be classified according to the risk of recent exposure based on differences in IFN-γ in TB1 and TB2 tubes that were higher than 0.6 IU·ml^−1^. QuantiFERON-TB gold plus is not able to distinguish between recent and remotely acquired tuberculosis infection, and it should not be used for that purpose in contact tuberculosis tracing.

## INTRODUCTION

Contact tracing is central to tuberculosis (TB) control and prevention by helping to identify and treat people who have been recently infected (1). The tuberculin skin test (TST) and, more recently, interferon gamma (IFN-γ) release assays (IGRAs) have been used to detect TB infection among contacts of TB cases. However, both tests have a poor ability to predict progression from latent to active TB, with the IGRAs being, at best, only slightly better than the TST (2–5). Presently, in contact tracing, except for converters, there is no method to confirm whether a positive IGRA result is due to recent exposure or exposure that occurred several years earlier. Consequently, all positive results are considered recent infections and all of them are given preventive therapy to avoid leaving high-risk subjects untreated.

In recent years, the importance of CD8^+^ T-cells in the immune response against Mycobacterium tuberculosis infection has been recognized (6, 7). It has been shown that the CD8^+^ T-cell response is higher in recent contacts of TB patients than in other groups (8). In this regard, the QuantiFERON-TB gold plus (QFT-plus), which includes an additional antigen tube (TB2) that can elicit IFN-γ production by both CD4^+^ and CD8^+^ T-cell responses, provides a surrogate marker of the CD8^+^ T-cell response (9). In two previous studies (10, 11), an antigen tube 2 minus antigen tube 1 (TB2−TB1) value >0.6 IU·ml^−1^ in contacts was associated with sleeping in the same room as index cases and with European origin (10), and a higher quantitative TB2 IFN-γ response was found in contacts living in the same room as index cases (11), data highly suggestive of newly acquired infection. However, these studies included small numbers of individuals with TB2−TB1 values >0.6 IU·ml^−1^, and they did not compare contacts with groups without risk of recent exposure.

In the present study, we investigated whether the QFT-plus could be used to identify individuals recently exposed to TB and thereby act as a surrogate marker for newly acquired infection. This was done by identifying TB2, TB1, and TB2−TB1 IFN-γ levels in individuals with different risks of recent TB exposure. The rationale was that if an association exists between differences in IFN-γ production in TB2 and TB1 and time elapsed since primary infection, the QFT-plus should be able to stratify the groups by their background exposure. Specifically, we anticipated a gradient in the proportions of individuals with TB2−TB1 IFN-γ values >0.6 IU·ml^−1^ across the groups (with the highest rates in contacts and lower rates in individuals without risk of recent TB exposure).

## RESULTS

In total, we included 686 adults with positive QFT-plus results, with 368 in the contact group, 229 in the patients with immune-mediated inflammatory diseases (IMID) group, and 89 in the asylum seekers or people from abroad (ASPFA) group. Their main characteristics are summarized in Table 1. Compared with the contact and IMID groups, the ASPFA group predominantly included men (49.5%, 57.2%, and 70.8%, respectively; *P* = 0.001) and its members were younger (median ages 44.6, 58.0, and 24.5 years, respectively; *P* < 0.001). Of note, 58.2% of contacts were from countries with <25 TB cases per 10^5^ population.

**TABLE 1:** Main characteristics of 686 individuals with positive QuantiFERON-TB gold plus results

Characteristic^*a*^	No. (%) or other value as indicated for^*b*^:			*P* value
	Contacts (*n* = 368)	IMID patients (*n* = 229)	ASPFA (*n* = 89)	
Gender				
Male	182 (49.5)	131 (57.2)	63 (70.8)	0.001
Female	186 (50.5)	98 (42.8)	26 (29.2)	
Age (yr)				
Mean (±SD)	45.4 (±16.9)	55.8 (±13.6)	28.5 (±11.9)	<0.001
Median (IQR)	44.6 (32.1–55.5)	58.0 (44.4–66.7)	24.5 (20.1–33.1)	<0.001
BCG vaccination status				
Yes	195 (53)	43 (18.8)	12 (13.5)	<0.001
No	90 (24.4)	28 (12.2)	17 (19.1)	
Unknown	83 (22.6)	158 (69.0)	60 (67.4)	
Incidence of TB in country of birth				
≥25 × 10^5^	154 (41.8)	17 (7.4)	83 (93.3)	<0.001
<25 × 10^5^	214 (58.2)	212 (92.6)	6 (6.7)	
Type of contact				
Close	202 (54.9)			
Frequent	104 (28.3)			
Sporadic	62 (16.8)			
QFT-plus positive at baseline				
Yes	353 (95.9)			
No	15 (4.1)			
Contact with a smear-positive case				
Yes	285 (77.4)			
No	75 (20.4)			
Unknown	8 (2.2)			
IMID				
≤10 mg PDN or equivalent within 8 wk				
Yes		53 (23.1)		
No		176 (76.9)		
DMARDs within 8 wk				
Yes		98 (42.8)		
No		131 (57.2)		

a BCG, bacillus Calmette-Guérin; DMARDs, disease-modifying antirheumatic drugs; IQR, interquartile range; PDN, prednisone; QFT-plus, QuantiFERON-TB gold plus; TB, tuberculosis.

b ASPFA, asylum seekers and people from abroad; IMID, immune-mediated inflammatory diseases.

### TB1 and TB2 interferon-γ responses.

**FIG 1:**
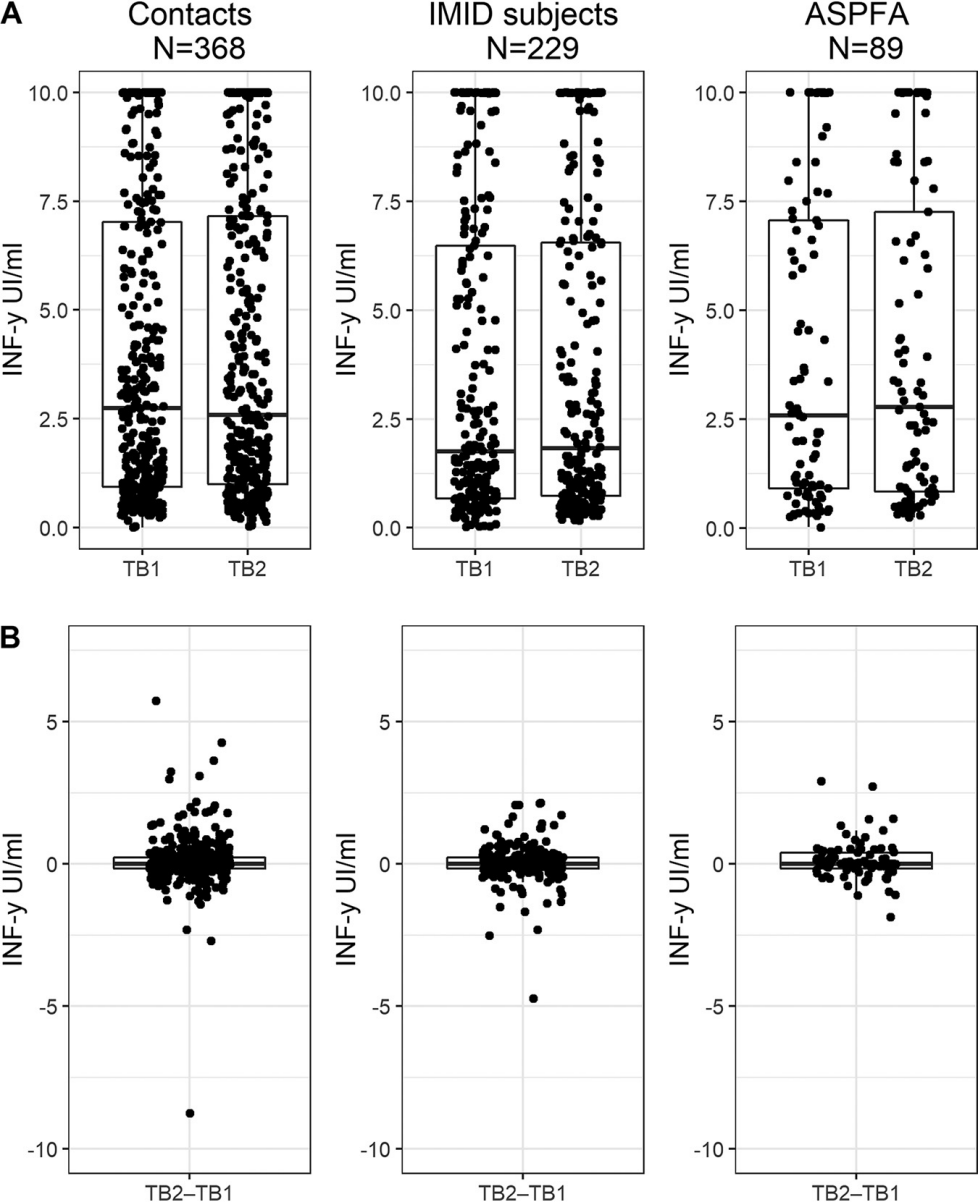
TB1−Nil and TB2−Nil (A) and TB2−TB1 (B) values for IFN-γ responses in the QuantiFERON-TB gold plus by group. Responses are shown using dot and box plots with whiskers, where the horizontal lines indicate the median values and the first and third quartiles. Within-group comparison (TB1−Nil and TB2−Nil): contacts, *P* = 0.083; IMID patients, *P* = 0.220; and ASPFA, *P* = 0.214. Between-group comparison: TB1−Nil, *P* = 0.084; TB2−Nil, *P* = 0.095; and TB2−TB1, *P* = 0.935. ASPFA, asylum seekers and people from abroad; IMID, immune-mediated inflammatory disease; TB1, antigen tube 1 of the QFT-plus; TB2, antigen tube 2 of the QFT-plus.

Respectively, the median IFN-γ concentrations for TB1 minus Nil (TB1−Nil) and TB2 minus Nil (TB2−Nil) were 2.74 and 2.58 IU·ml^−1^ in the contact group (*P* = 0.083), 1.76 and 1.83 IU·ml^−1^ in the IMID group (*P* = 0.220), and 2.59 and 2.78 IU·ml^−1^ in the ASPFA group (*P* = 0.214). There were no significant differences for TB1−Nil (*P* = 0.084) or TB2−Nil (*P* = 0.095) between groups (Fig. 1 and Table S1 in the supplemental material).

### TB1 and TB2 interferon-γ response among contacts.

**FIG 2:**
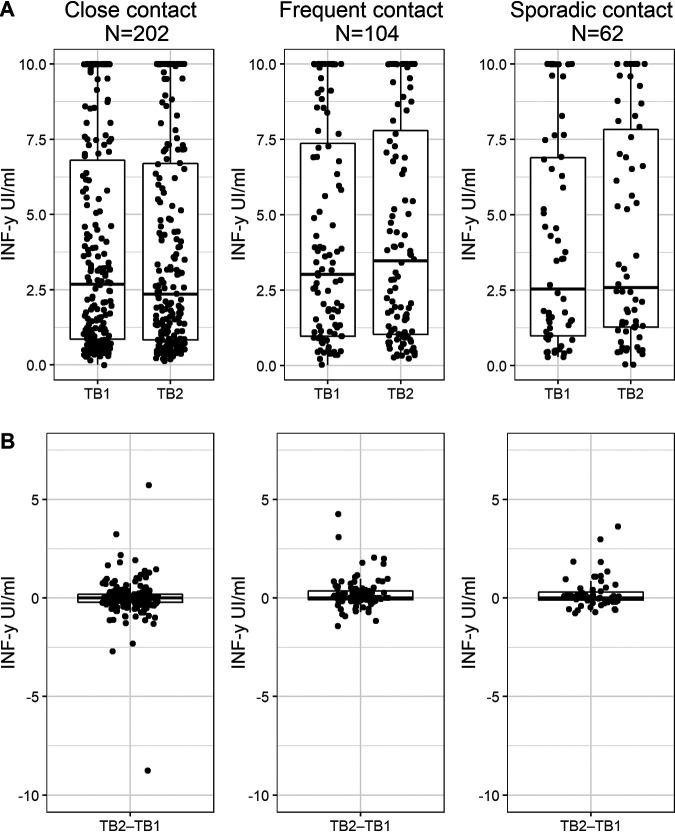
TB1−Nil and TB2−Nil (A) and TB2−TB1 (B) values for IFN-γ responses in the QuantiFERON-TB gold plus by extent of exposure. Responses are shown using dot and box plots with whiskers, where the horizontal lines indicate the median values and the first and third quartiles. Within-group comparison (TB1−Nil and TB2−Nil): close contacts, *P* = 0.785; frequent contacts, *P* = 0.016; and sporadic contacts, *P* = 0.118. Between-group comparison: TB1−Nil, *P* = 0.646; TB2−Nil, *P* = 0.371; and TB2−TB1, *P* = 0.053. TB1, antigen tube 1 of the QFT-plus; TB2, antigen tube 2 of the QFT-plus.

When we restricted the analysis to the 368 contacts, the respective median IFN-γ concentrations for TB1−Nil and TB2−Nil were 2.68 and 2.36 IU·ml^−1^ for close contacts (*P* = 0.785), 3.03 and 3.47 IU·ml^−1^ for frequent contacts (*P* = 0.016), and 2.54 and 2.58 IU·ml^−1^ for sporadic contacts (*P* = 0.118). No significant differences were observed for TB1−Nil (*P* = 0.646) or TB2−Nil (*P* = 0.371) when comparing these three subgroups (Fig. 2 and Table S2).

**FIG 3:**
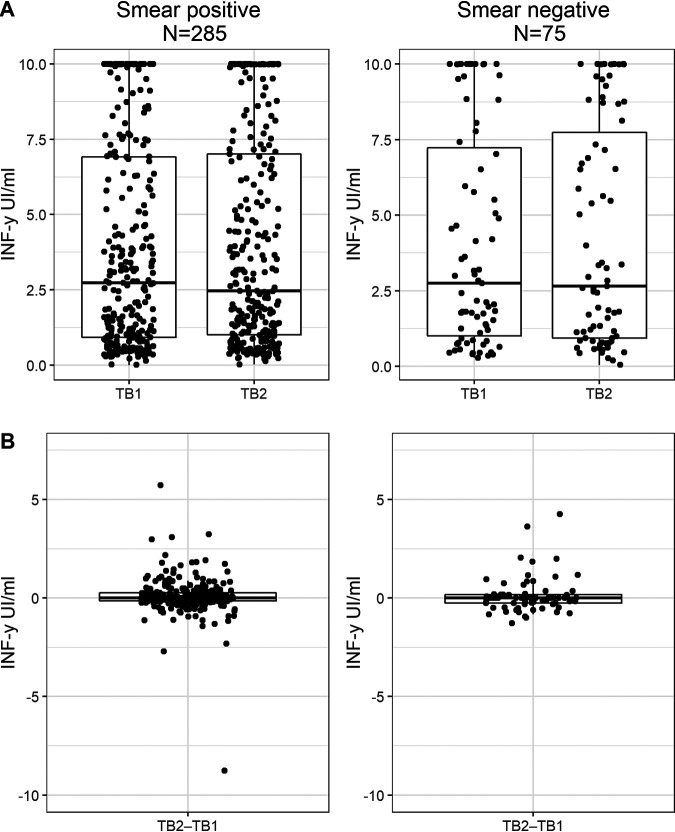
TB1−Nil and TB2−Nil (A) and TB2−TB1 (B) values for IFN-γ responses in the QuantiFERON-TB gold plus by sputum smear microscopy result of the index case. Responses are shown using dot and box plots with whiskers, where the horizontal lines indicate the median values and the first and third quartiles. Within-group comparison (TB1−Nil and TB2−Nil): *P* = 0.031 for smear positive and *P* = 0.906 for smear negative. Between-group comparison: *P* = 0.655 for TB1−Nil, *P* = 0.730 for TB2−Nil, and *P* = 0.457 for TB2−TB1. TB1, antigen tube 1 of the QFT-plus; TB2, antigen tube 2 of the QFT-plus.

Next, we analyzed the 360 contacts for whom the sputum smear microscopy result of the index case was known. The median IFN-γ concentrations for TB1−Nil and TB2−Nil were 2.73 and 2.47 IU·ml^−1^ among smear-positive contacts (*P* = 0.031), compared with 2.75 and 2.65 IU·ml^−1^ among smear-negative contacts (*P* = 0.906). There were no significant differences for TB1−Nil (*P* = 0.655) or TB2−Nil (*P* = 0.730) when comparing these two groups (Fig. 3).

Given that the conversion of QFT-plus from a negative to a positive result in contact tracing denotes newly acquired infection, we also looked at the IFN-γ concentrations among the 15 contacts that showed conversion. The median IFN-γ concentrations were 1.50 IU·ml^−1^ for TB1−Nil and 1.12 IU·ml^−1^ for TB2−Nil. Among these, 11 contacts showed conversion in both tubes (8 presented conversion outside the borderline range), 2 showed conversion in only TB1, and 2 showed conversion in only TB2 (all four within the borderline range). Although 8 of the 15 had higher IFN-γ concentrations in the TB2 tube than in the TB1 tube at the second test, the TB2−TB1 was >0.6 IU·ml^−1^ in only one case (Table S3).

### TB2−TB1 value >0.6 IU·ml^−1^ as a predictor of greater risk of recent exposure.

The proportion of cases with IFN-γ concentrations higher in TB2 than in TB1 (TB2>TB1) was 44.6% (43.5% in the contact group, 46.3% in the IMID group, and 44.9% in the ASPFA group; *P* = 0.796). The respective proportions among the 368 contacts were 39.1% of close contacts, 50.0% of frequent contacts, and 46.8% of sporadic contacts (*P* = 0.162).

When using the >0.6 IU·ml^−1^ criterion (TB2−TB1 > 0.6 IU·ml^−1^), these proportions decreased (13.6% in the contact group, 10.9% in the IMID group, and 11.2% in the ASPFA group; *P* = 0.591). The proportions also decreased when we analyzed the contact subgroups (11.4% among close contacts, 15.4% among frequent contacts, and 17.7% among sporadic contacts; *P* = 0.362).

A TB2−TB1 value >0.6 IU·ml^−1^ was not associated with a greater risk of recent TB exposure. Versus the contact group, the odds ratios (ORs) were 0.78 (95% confidence interval [CI], 0.47 to 1.30) for the IMID group and 0.81 (95% CI, 0.39 to 1.66) for the ASPFA group in the univariate regression analysis. This lack of association remained after adjusting for covariates (i.e., age, gender, incidence of TB in the country of birth [<25 × 10^5^ or ≥25 × 10^5^], and European origin) (Table 2). Similarly, a TB2−TB1 value >0.6 IU·ml^−1^ was not associated with a closer exposure. Versus the close contact subgroup, ORs were 1.47 (95% CI, 0.74 to 2.94) for frequent and 1.77 (95% CI, 0.81 to 3.89) for sporadic subgroups in the univariate regression analysis. This lack of association also remained after the corresponding adjusted analyses for the same variables plus the sputum smear result of the index case (Table 2).

**TABLE 2:** Utility of a TB2−TB1 IFN-γ value >0.6 IU·ml^−1^ for predicting a higher likelihood of recent tuberculosis infection^*a*^

Variable	No. with QFT-plus (+)	Crude analysis		Adjusted analysis^*b*^	
		OR (95% CI)	*P* value	OR (95% CI)	*P* value
Whole cohort (*n* = 686)					
Group (likelihood of recent infection)					
Contacts (high)	368	1		1	
IMID patients (low)	229	0.78 (0.47–1.30)	0.339	0.71 (0.31–1.61)	0.406
ASPFA (indeterminate)	89	0.81 (0.39–1.66)	0.556	0.86 (0.49–1.52)	0.604

Contacts with known sputum smear result for the index case (*n* = 360)					
Type of contact					
Close	201	1		1	
Frequent	100	1.47 (0.74–2.94)	0.269	1.29 (0.63–2.62)	0.490
Sporadic	59	1.77 (0.81–3.89)	0.153	1.55 (0.67–3.60)	0.307

a ASPFA, asylum seekers or people from abroad; CI, confidence interval; IMID, immune-mediated inflammatory diseases; OR, odds ratio; TB1, antigen tube 1 of the QFT-plus; TB2, antigen tube 2 of the QFT-plus.

b Adjusted for age, gender, incidence of tuberculosis in the country of birth (< or ≥25 × 10^5^), and European origin (yes or no) for the whole cohort and contacts, and also adjusted for the sputum smear result of the index case (positive or negative acid-fast bacillus stain) for contacts.

## DISCUSSION

The introduction of the QFT-plus test has been accompanied by the expectation that this would allow newly acquired TB infection to be distinguished from remotely acquired TB infection. However, in this study, we could not validate previous findings suggesting that a TB2−TB1 value >0.6 IU·ml^−1^ served as a proxy marker for recent infection. In one study (11), 14 of 105 (13.3%) infected contacts had a TB2−TB1 value >0.6 IU·ml^−1^, and the quantitative TB2 IFN-γ response was higher in contacts living in the same room as index cases than in other contacts. In an Italian study performed in a cohort of 119 TB contacts, 18 of 68 (26.5%) QFT-plus-positive contacts had a TB2−TB1 value >0.6 IU·ml^−1^. This revealed an independently positive association between a TB2−TB1 value >0.6 IU·ml^−1^ and both European origin and sleeping in the same room as the index case, consistent with newly acquired infection from the index case (10). However, the chief limitation of these two prior studies was the absence of comparison between contacts and groups with low or zero risk of recent exposure. Indeed, coupled with the small numbers of individuals with TB2−TB1 values >0.6 IU·ml^−1^ (14 and 18 in each study, respectively), this was a major limitation on their ability to obtain definitive results about a hypothetical association between TB2−TB1 and risk of recent TB exposure. A laboratory-based data study (12) found that contacts and individuals with periodic checks by occupational health services had a higher proportion (33%) of TB2−TB1 values >0.6 IU·ml^−1^ than did patients tested before immunosuppressant therapy (11%). However, the consistency of the results was again limited by the small number of participants with a TB2−TB1 value >0.6 (18 contacts and 2 patients tested before immunosuppressant therapy).

In our study, to provide a contrast with data for individuals at high risk of recent exposure, we included a control group of individuals without known risk of recent exposure (IMID group) and another group of individuals with indeterminate risk (ASPFA group). We included the ASPFA group because it is representative of those commonly screened for TB infection in Europe, and as such, the findings could have real-life implications for clinical management. Our results show that samples from individuals with high, low, and indeterminate risk of recent exposure behave in the same manner in terms of the IFN-γ response and differences in TB1 and TB2 tubes. Two additional analyses support this observation. First, only 1 of the 15 converters (which supposedly indicates recently acquired infection) had a TB2−TB1 value >0.6 IU·ml^−1^, and second, there was no association between the smear status of the index case and a TB2−TB1 value >0.6 IU·ml^−1^. Given that a positive sputum smear in the index case implies a greater risk of transmission, our findings support even further the lack of correlation between a higher IFN-γ response in the TB2 tube and recent TB exposure.

Consistent with our data, a cross-sectional study of 31 recent contacts with positive QFT-plus results (from a total of 412 TB contacts) failed to show a distinct CD8^+^ T-cell response (13). In another prospective study of 492 TB contacts that assessed the QFT-plus value for predicting incident TB, the receiver operating characteristic curves were similar for the TB1 and TB2 results (14). Therefore, a difference between the TB2 and TB1 results (as a proxy for the CD8^+^ T-cell response) failed to discriminate progressors from nonprogressors, indirectly providing evidence of a lack of discriminative value for recent TB exposure (14).

To the best of our knowledge, this is the largest study to have investigated the difference in IFN-γ response between TB1 and TB2 QFT-plus tubes in individuals with different risks of recent TB exposure. Contrary to previous studies, our results argue against the potential clinical utility of a TB2−TB1 value >0.6 IU·ml^−1^ as a proxy for recent TB infection.

This study has limitations that deserve comment. First, the retrospective design may have jeopardized the accuracy of data on contact status and immunosuppression in the IMID group, which may have led to some cases being misclassified. Second, the actual hours and places of exposure to the index case were only available in a small number of cases, and these data were discarded for the analyses. This limitation was palliated, at least in part, by the fact that the concentric circle approach already accounts for the place of exposure and stratifies contacts for more/less than 6 h and for daily versus not daily. Third, the optimal control group would have been healthy individuals with positive QFT-plus results and no evidence of recent contact. However, it was not feasible to identify such patients because screening is not routinely performed in otherwise healthy people with no risk of developing active TB.

In conclusion, a difference of >0.6 IU·ml^−1^ in the TB2−TB1 value was not associated with a higher risk of recent TB exposure, which puts into question the potential clinical utility of this measure as a proxy that can identify recently acquired TB infection.

## MATERIALS AND METHODS

### Study design and participants.

We conducted a retrospective multicenter study to evaluate the IFN-γ responses in QFT-plus tubes, including the differences between the TB1 and TB2 tubes (i.e., the TB2−TB1 value), in people who tested positive for TB infection between 1 June 2016 and 31 May 2018. The study was conducted at 19 centers in 7 countries (11 hospitals in Spain, 2 in Sweden, 2 in Croatia, and 1 each in Chile, Turkey, Serbia, and Italy).

We included individuals ≥18 years of age who were (i) assessed as a part of a TB contact investigation, (ii) screened for TB infection prior to therapy with biologic agents because of immune-mediated inflammatory diseases (IMID), or (iii) asylum seekers or people from abroad (ASPFA). We only included contacts of microbiologically confirmed pulmonary TB patients. The extent of exposure was defined, according to the concentric circles approach (15), as close when the contact was ≥6 h daily, frequent when the contact was <6 h daily, and sporadic when it was not daily. For patients screened before biological therapy, we only included those without known recent exposure to a patient with TB. We excluded individuals with HIV infection, advanced chronic liver disease, end-stage kidney disease, solid organ or hematopoietic stem cell transplantation, corticosteroid treatment (>10 mg/day of prednisone or equivalent), biologic agents, or chemotherapy for cancer in the 8 weeks before testing or with suspicion of active TB.

According to the risk of recent TB exposure, participants were considered (i) high risk, which included contacts of TB cases, (ii) low risk, which included patients screened before therapy with biologics, and (iii) indeterminate risk, which included ASPFA.

Conversion of the QFT-plus test was defined has having a positive test that followed a negative test performed 8 to 12 weeks apart, according to the manufacturer’s instructions (16). A more conservative definition of conversion (outside the borderline range) (17) was established as an increase in the IFN-γ concentration from <0.20 IU·ml^−1^ to >0.99 IU·ml^−1^ in the TB1, TB2, or both tubes.

### Data collection and analysis.

We collected data on demographics, reasons for testing, bacillus Calmette-Guérin (Mycobacterium bovis BCG) vaccination status, and QFT-plus results. Study data were collected and managed using REDCap (Research Electronic Data Capture) tools hosted at Bellvitge Biomedical Research Institute (IDIBELL) (18). Due to the exploratory aim of the study, no formal sample size calculation was determined based on a single hypothesis test. The final number of included participants was defined by the available participants in each center.

The data analysis proceeded as follows. First, we compared TB1 and TB2 IFN-γ concentrations within and between recent exposure risk groups (i.e., high, low, and indeterminate) and within and between each contact type (i.e., close, frequent, and sporadic). Second, we compared the proportions of cases in which IFN-γ production was higher in TB2 than in TB1 between groups by risk of recent exposure and type of contact. Finally, we compared the TB2−TB1 values between the same groups. Given that the intrinsic variability of the QFT-plus could produce differences between the two tubes, we reduced this bias by treating any difference in TB2−TB1 values >0.6 IU·ml^−1^ as a real difference. We chose this cutoff (TB2−TB1 > 0.6 IU·ml^−1^) because this has been considered the threshold for CD8^+^ T-cell response and, by using this, some studies have shown associations between TB2−TB1 > 0.6 IU·ml^−1^ and exposure intensity, proximity to the index case, and proximity to time of infection (10–12).

We performed Mann-Whitney tests for comparisons between two groups and Kruskal-Wallis tests for comparisons among more than two groups. The Wilcoxon matched-pairs signed-rank test was used to compare two variables within the same group. We assessed the association of the TB2−TB1 value with a higher probability of recent infection by crude and adjusted logistic regression analysis. First, we assessed the association of a TB2−TB1 value >0.6 IU·ml^−1^ and risk of recent exposure by univariate regression analysis, both in the whole cohort (contacts, IMID patients, and ASPFA) and in the contact subgroups (close, frequent, and sporadic), and second, we adjusted for variables potentially associated with changes in TB2−TB1 results (age, gender, incidence of TB in the country of birth, and European origin, for the whole cohort and contacts, and also adjusted for the sputum smear of the index case), regardless of their significance in the univariate analysis. All reported *P* values were calculated with statistical significance set at a *P* value of less than 0.05. Odds ratios (ORs) and 95% confidence intervals (CIs) are reported. Statistical analyses were performed using R version 3.5.0 for Windows (19).

### Ethics.

The study received ethical clearance from the Ethics Committees of all participating centers. The confidentiality of data was protected according to National and European Protection Data law (e.g., General Data Protection Regulation; EU, 2016/679).

### Data availability.

Data are fully available at the REDCap tools hosted at Bellvitge Biomedical Research Institute (IDIBELL) upon request to the principal investigator and corresponding author Miguel Santin.
