# Supplementary material for: Identification of Recent Tuberculosis Exposure Using QuantiFERON-TB Gold Plus, a Multicenter Study
Source: Microbiol Spectr. 2021 Nov 10;9(3):e00972-21. doi: 10.1128/Spectrum.00972-21 (PMC8579846; doi:10.1128/Spectrum.00972-21)
Supplement: SUPPLEMENTAL FILE 1 — Supplemental material. Download SPECTRUM00972-21_Supp_1_seq7.pdf, PDF file, 0.2 MB [file spectrum00972-21_supp_1_seq7.pdf]

TABLE S1. Interferon-γ production in QuantiFERON-TB Gold Plus tubes by study group

	**Contacts**<br><br>**N = 368**	**IMID patients**<br><br>**N = 229**	**ASPFA**<br><br>**N = 89**	***p***
**TB1–NIL IU•mL^{-1},** Median [Q1;Q3]	2.74 [0.94;7.02]	1.76 [0.67;6.48]	2.59 [0.91;7.07]	0.084
**TB2–NIL IU•mL^{-1},** Median [Q1;Q3]	2.58 [1.00;7.16]	1.83 [0.74;6.56]	2.78 [0.84;7.26]	0.095
p*	0.083	0.220	0.214	
**TB2–TB1** IU•mL^{-1}**, Median [Q1;Q3]	0.00 [-0.16;0.23]	0.00 [-0.16;0.23]	0.00 [-0.17;0.39]	0.935
**Mitogen,** Median [Q1;Q3]	10.0 [10.0;10.0]	10.0 [9.70;10.0]	10.0 [7.86;10.0]	0.001

Abbreviations: ASPFA = Asylum seekers and people from abroad; IMID = Immune-mediated inflammatory diseases; TB1 = Antigen tube 1 of the QFT-Plus; TB2 = Antigen tube 2 of the QFT-Plus.

*Comparison between TB1–Nil and TB2–Nil within each group

**TB1 and TB2 minus Nil

TABLE S2. Interferon-γ production in QuantiFERON-TB Gold Plus tubes by the closeness of the exposure

	**Close**<br><br>**N = 202**	**Frequent**<br><br>**N = 104**	**Sporadic**<br><br>**N = 62**	***p***
**TB1–Nil IU•mL^{-1}**, Median [Q1;Q3]	2.68 [0.86;6.80]	3.03 [0.97;7.38]	2.54 [0.99;6.90]	0.646
**TB2–Nil IU•mL^{-1}**, Median [Q1;Q3]	2.36 [0.84;6.70]	3.47 [1.04;7.80]	2.58 [1.27;7.83]	0.371
p*	0.785	0.016	0.118	
**TB2–TB1** IU•mL^{-1}**, Median [Q1;Q3]	0.00 [-0.23;0.20]	0.00 [-0.08;0.35]	0.00 [-0.11;0.31]	0.053
**Mitogen**, Median [Q1;Q3]	10.0 [10.0;10.0]	10.0 [9.98;10.0]	10.0 [9.75;10.0]	0.953

Abbreviations: IFN = interferon; TB1 = Antigen tube 1 of the QFT-Plus; TB2 = Antigen tube 2 of the QFT-Plus

The analysis included 368 individuals with tuberculosis contact.

*Comparison between TB1–Nil and TB2–Nil within each group

**TB1 and TB2 minus Nil

TABLE S3. Interferon-γ production in QuantiFERON-TB Gold Plus tubes among the 15 cases with conversion

	Negative QFT-Plus		Positive QFT-Plus				
	TB1–NiL IU•mL^{-1}	TB2–Nil IU•mL^{-1}	TB1–NiL IU•mL^{-1}	TB2–Nil IU•mL^{-1}	TB2–TB1* IU•mL^{-1}	TB2 >TB1*	TB2–TB1* >0.6
**1**	0.25	0.22	0.29	0.49	0.2	Yes	No
**2**	0	0	0.47	0.32	-0.15	No	No
**3**	0.14	0.09	0.45	0.33	-0.12	No	No
**4**	0.01	0	6.93	6.93	0	No	No
**5**	0.01	0.01	1.65	1.96	0.31	Yes	No
**6**	0.03	0.03	1.5	1.12	-0.38	No	No
**7**	0.03	0	2.45	4.1	1.65	Yes	Yes
**8**	0	0	2.08	1.86	-0.22	No	No
**9**	0.07	0.03	4.3	4.42	0.12	Yes	No
**10**	-0.49	0.11	0.28	0.41	0.13	Yes	No
**11**	0.3	0.23	0.46	0.61	0.15	Yes	No
**12**	0.31	0.3	3.12	3.5	0.38	Yes	No
**13**	0.16	0.25	1.61	1.35	-0.26	No	No
**14**	0.28	0.33	0.77	0.46	-0.31	No	No

**15**	0.07	0	0.45	0.68	0.23	Yes	No

Abbreviations: TB1 = Antigen tube 1 of the QFT-Plus; TB2 = Antigen tube 2 of the QFT-Plus

*TB1 and TB2 minus Nil
